# An Effect of Cyclosporin A in a Treatment of Temporal Bone Defect Using hBM-MSCs

**DOI:** 10.3390/biomedicines10112918

**Published:** 2022-11-14

**Authors:** Lukas Skoloudik, Viktor Chrobok, Jan Laco, Jana Dedkova, Daniel Diaz Garcia, Stanislav Filip

**Affiliations:** 1 Department of Otorhinolaryngology and Head and Neck Surgery, University Hospital Hradec Kralove, Faculty of Medicine in Hradec Kralove, Charles University, 500 03 Hradec Králové, Czech Republic; 2 The Fingerland Department of Pathology, University Hospital Hradec Kralove, Faculty of Medicine in Hradec Kralove, Charles University, 500 03 Hradec Králové, Czech Republic; 3 Department of Radiology, University Hospital Hradec Kralove, 500 05 Hradec Králové, Czech Republic; 4 Department of Pharmacology, Faculty of Medicine in Hradec Kralove, Charles University, 500 03 Hradec Králové, Czech Republic; 5 Department of Oncology and Radiotherapy, Faculty of Medicine Hradec Kralove, Charles University, 500 03 Hradec Králové, Czech Republic

**Keywords:** temporal bone, mesenchymal stem cells, cyclosporin A, osteogenesis, cholesteatoma

## Abstract

*Background.* The treatment of middle ear cholesteatoma requires surgical treatment and the reconstruction of the temporal bone, which represents an ongoing problem. Otologists have focused on the research of materials allowing an airy middle ear and the preservation of hearing function to reconstruct the temporal bone. *Methods.* This study evaluated the effect of cyclosporin A (CsA) and a combined biomaterial in the healing process of postoperative temporal bone defects in an animal model. Cultured human Bone Marrow Mesenchymal Stromal Cells (hBM-MSCs) were mixed with hydroxyapatite (Cem-Ostetic^®^), and subsequently applied as a bone substitute after middle ear surgery, showing that the therapeutic potential of hBM-MSCs associated with bone regeneration and replacement is directly influenced by CsA, confirming that it promotes the survival of MSCs in vivo. *Results.* The therapeutic efficacy of the combination of MSCs with CsA is greater than the sole application of MSCs in a hydroxyapatite carrier. *Conclusion.* The reconstruction of a temporal bone defect using hBM-MSCs requires an immunosuppressant to improve the results of treatment.

## 1. Introduction

Cholesteatoma is characterized by the keratinization of the squamous epithelium in the middle ear, usually beginning in the edge of the eardrum, damaging the tympanic cavity and mastoid bone. Cholesteatoma spreads in an aggressive manner, eroding the bone and often leading to conductive hearing loss through the destruction of the middle ear ossicles. Bone erosion caused by cholesteatoma can also lead to more serious complications, such as facial nerve palsy. The infection can reach the inner ear, causing sensorineural hearing loss, deafness, and dizziness. Nearly 7.5% of patients suffering from chronic middle ear disease with cholesteatoma developed intracranial complications such as meningitis, sigmoid sinus thrombophlebitis, and brain abscess [1,2].

Although these complications are rare, they are still serious and can be prevented by removing the cholesteatoma. Occasionally, middle ear cholesteatoma surgery requires a canal wall down (CWD) mastoidectomy. In this approach, the mastoid cavity is widely connected with the external ear canal. However, the ear canal loses its self-cleaning ability, and the exteriorized mastoid cavity becomes the target of chronic infections. These side effects precede the major drawbacks of CWD, such as recurrent ear discharge, ill-fitting hearing aids, bathing restrictions, and regular follow-up visits to clean debris from the mastoid cavity. Some of these cavity problems have been diminished using an obliteration technique, which only reduces the size of the mastoid cavity. Therefore, the current challenge lies in the closure of the mastoid cavity and the reconstruction of the bone barrier between the mastoid cavity and the ear canal [3,4].

Otologists have focused on researching temporal bone reconstruction materials enabling an airy middle ear and preserving hearing function. Bone reconstruction allows the use of mesenchymal stem cells (MSCs) [5]. These cells are readily available since they can be found in the bone marrow, adipose tissue, umbilical cord blood, placenta, dental pulp, skin, heart, and spleen [6].

The question is whether the use of MSCs needs an immunosuppressive therapy. Immunosuppressant drugs, e.g., cyclosporin A (CsA), are indicated during the transplantation to prevent graft-versus-host disease (GVHD) [7,8]. However, MSCs do not require this treatment because of their own immunosuppressant capacity [9,10].

In previous experiments, we focused on the reparative effect of xenogeneic hBM-MSCs loaded in a hydroxyapatite scaffold (Cem-Ostetic^®^, Berkeley Advanced Biomaterials, Inc., Berkeley, CA, USA) with CsA immunosuppressive treatment. These experiments confirmed the safe use of this procedure, as verified by the preservation of good hearing function [11]. With this in consideration, the question arose on the effect of CsA during MSCs reconstruction of the temporal bone. 

## 2. Materials and Methods

### 2.1. Animals

Eighteen guinea pigs were used for this study (male, 220–420 grams in weight). The animals were quarantined for two weeks before the experiment. The animals were housed with access to food and water ad libitum. Animal care followed the European Community Council and Czech Republic Directive guidelines (#86/609/EEC), and all experiments were performed with the approval of the Ethics Committee of the University Hospital and Medical Faculty Hradec Kralove (authorization # 42013). All animals had normal eardrums without middle ear discharge at the beginning of the experiments.

### 2.2. Experimental Design

**Figure 1:**
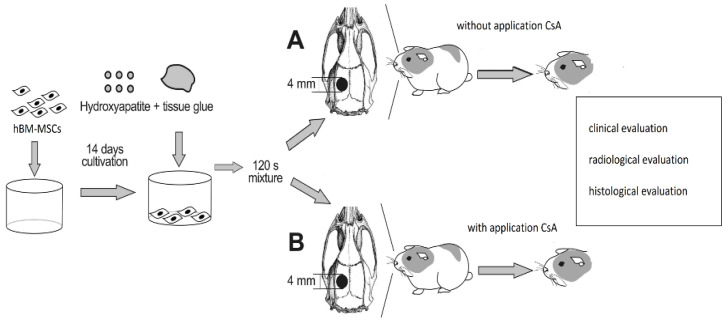
Schematic diagram of the experimental protocol. The biomaterial loaded with hBM-MSCs and implanted into the bone defects of guinea pigs was analyzed after two months of surgery. Group A is without application of CsA and Group B is with application of CsA. Various clinical, radiological, and histological parameters were evaluated.

The animals were divided in two groups: A (n = 9), animals without application CsA, and B (n = 9), animals with CsA (Figure 1).

All animals were implanted with the osteoconductive biomaterial Cem-Ostetic^®^ (Berkeley Advanced Biomaterials, Inc., Berkley, CA, USA) loaded with hBM-MSCs (MSC 3P suspended in 1.5 mL of Ringer’s solution stabilized human albumin; Bioinova, Ltd., Prague, Czech Republic). Following surgery, group B was treated with a 10 mg/kg dose of cyclosporine A on a daily basis (CSA; Sandimmune Neoral, Novartis, Germany). The animals were evaluated at 60 ± 2 days after the implantation of the composed biomaterial. At the end of the experiment, the animals were euthanized with 100 mg/kg ketamine hydrochloride i.p. (Gedeon-Richter, Hungary) and 10 mg/kg of xylazine hydrochloride i.p. (Interchemie Gasternory, Holland) followed by transcardial perfusion with 10% formaldehyde (Sigma-Aldrich; St. Louis, MO, USA).

### 2.3. Surgery

Guinea pigs were anesthetized with 50 mg/kg ketamine hydrochloride (Gedeon-Richter, Budapest, Hungary) and 5 mg/kg xylazine hydrochloride i.m. (Interchemie Gasternory, Holland). The left tympanic bulla was exposed using an anterior surgical approach (submandibular skin incision) and perforated on the anterior surface using Karl Storz Fisch manual perforator (Ø = 0.8 mm). The bony defect was enlarged with a microdissector and small forceps to Ø = 4 mm. The middle ear ossicles and the tympanic membrane were preserved. The bony defect was covered with the biomaterial loaded with MSCs. The wound was closed with a single layer of 3-0 Safil suture. The animals were treated with 4 mg/kg carprofen (analgesic) (Janssen Pharmaceutica NV, CCPC, Belgium) and 10 mg/kg enrofloxacine (antibiotics) i.m. (eBioscience, San Diego, CA, USA) at the end of the surgery.

### 2.4. Cell Preparation

Bone marrow was obtained from the iliac crest of healthy donors with informed consent and processed in a clean room facility (Bioinova, Ltd., Prague, Czech Republic). In brief, the bone marrow was mixed with Gelofusine^®^ (B. Braun, Melsungen AG, Germany) and the mononuclear fraction collected and cultured on plastic flasks at a density of 70,000–140,000 cells/cm^2^ (TPP Techno Plastic Products AG; Trasadingen, Switzerland). Non-adherent cells were removed after 24 and 48 h by replacing the media. The adherent cells were further incubated at 37 °C in a humidified atmosphere containing 5% CO_2_ in enriched MEMAlpha (Lonza Walkersville Inc., Walkersville, MD, USA) media containing 5% platelet lysate (Bioinova, Ltd., Czech Republic) and 10 μg/mL gentamicin (Gentamicin Lek^®^; Lek Pharmaceuticals, Ljubljana, Slovenia). The media were replaced twice per week. MSCs were identified according to their spindle-shaped morphology and adherence capacity, with their phenotype later confirmed through flow cytometry (FACSAria; BD Biosciences, San Jose, CA, USA) using the markers lin^-^/CD105^+^/CD73^+^/CD90^+^. To determine the differentiation potential of these MSCs, the cells were differentiated into osteogenic, chondrogenic, and adipogenic lineages using standard differentiation media. Cell viability (≥95%) was evaluated using trypan blue staining, and the cultures were tested for bacterial and/or fungal contamination. Afterwards, a cell suspension containing 20 × 10^6^ of hBM-MSCs 3P (3rd passage) was greenlit for transplantation into temporal bone defects in guinea pigs. 

### 2.5. Scaffold Preparation

Hydroxyapatite (Cem-Ostetic^®^; Berkeley Advanced Biomaterials, Berkley, CA, USA) was used as an osteoconductive material for hMSCs. Thanks to its chemical similarity with organic bone (Ca_10_ (PO_4_)_6_ (OH)_2_), the material has excellent biocompatibility and was tested successfully as a suitable scaffold for osteogenic cells. A 1 cm^3^ section of synthetic bone graft was covered with 100 mg of tissue glue (Tisseel Lyo, Baxter Czech; Prague, Czech Republic) and seeded with 1.6 × 10^6^ hBM-MSCs. Each osteogenic scaffold was implanted according to their suitability to the created bone defect.

### 2.6. Histopathological Evaluation

The temporal bone samples were fixed in 10% neutral formalin (Sigma-Aldrich; St. Louis, MO, USA) for 24 h and gently decalcified in 5% formic acid (Sigma-Aldrich; St. Louis, MO, USA) for 5 days. The remaining tissue was embedded in paraffin, cut into 4 μm-thick sections, and stained with haematoxylin (Sigma-Aldrich; St. Louis, MO, USA) and eosin (Junsei Chemicals, Tokyo, Japan); or H&E and Masson’s trichrome (TRI) (MT, Sigma-Aldrich; St. Louis, MO, USA). In addition, the sections were stained with chloroacetate esterase (CHAE) (MT, Sigma-Aldrich; St. Louis, MO, USA) following the standard protocol. The structural changes in the middle and inner ear were evaluated in the H&E sections. For the immunohistochemical analysis, the sections were mounted on slides coated with 3-aminopropyltriethoxy-silane, deparaffinized in xylene, and rehydrated in descending grades of ethanol (70–100%) (all reagents: Sigma-Aldrich; St. Louis, MO, USA). The following antibodies were used: anti-CD3 (polyclonal, 1:200), anti-CD20 (L26, 1:300), anti-CD31 (JC70A, 1:50), and anti-TRAP (ab58008, 1:750); all antibodies were purchased from Dako (Dako Denmark A/S, Glostrup, Denmark), except for TRAP (Abcam, Cambridge, United Kingdom). Antigen retrieval was performed in a microwave vacuum histoprocessor RHS 1 (Milestone; Sorisole, Italy) at 97 °C/pH 6.0 for 4 min. Endogenous peroxidase activity was quenched using 3% hydrogen peroxide (MT; Sigma-Aldrich; St. Louis, MO, USA). After incubation with the antibodies, the sections were subjected to EnVision+ Dual Link System-HRP (Dako Denmark A/S, Glostrup, Denmark). Finally, the sections were stained with 3-3′-diaminobenzidine (DAB) and counterstained with H&E. Immunostaining was performed in an immunostainer BenchMark Ultra (Roche, Basel, Switzerland), using Ultra View Universal DAB Detection Kit and Bluing Reagent as the visualization reagent and chromogen (all reagents: Roche, Basel, Switzerland). Appropriate positive and negative controls were used. All the samples were evaluated by a pathologist in a blind study. New bone formation and inflammatory response were evaluated in the grafted area using a light microscope, making an absolute count of CHAE-positive neutrophils, eosinophils, CD3^+^ T-lymphocytes, and CD20^+^ B-lymphocytes. A total of three high-power fields (HPFs) (10× and 40×; A = 0.152 mm^2^) were used for the statistical analysis. The presence of TRAP-positive cells was also recorded but not quantified, as there was non-specific background staining, making accurate counting impossible. New bone formation was evaluated using the NIS Elements AR 3.0 program, calculated as the ratio:

$$\frac{\mathrm{total}\;\mathrm{area}\;\mathrm{of}\;\mathrm{newly}\;\mathrm{formed}\;\mathrm{bone}\;\mathrm{trabeculae}\;}{\mathrm{total}\;\mathrm{area}\;\mathrm{of}\;\mathrm{the}\;\mathrm{implantation}\;\mathrm{zone}\;}$$

### 2.7. Radiological Evaluation

The radiological examination was performed using a 128-row multi-detector Computed Tomography (CT; SIEMENS SOMATOM Definition AS/AS_+_) with a HRCT (high-resolution computed tomography) reconstruction algorithm. Source data collimation was of 0.625 mm, table feed was 4 mm/s, coronal plane reconstructions were obtained, slice thickness was 0.4 mm, FOV was 55 × 55 mm, matrix was 512 × 512, and images were evaluated in window center 700 HU and window width 4000 HU.

### 2.8. Statistical Analysis

The results obtained from an Equal-Variance *t*-test (software NCSS9) are expressed as mean ± SEM, where *p ≤* 0.05 was considered significant. The hearing tests, evaluated by distortion-product otoacoustic emission (DPOAE), were analyzed through a one-way ANOVA and a Bonferroni post hoc test (*p* ≤ 0.05). All of the analyses were performed by a certified statistician.

## 3. Results

### 3.1. Clinical Evaluation

All of the included animals in this study (n = 18) survived for 60 months without otitis media nor postoperative vestibulopathy. No other signs of relevant postoperative complications were observed, and wound healing was achieved successfully in all of the animals. Concerning group A (without application CsA), macroscopic exploration of the tympanic bulla showed that the operative defect was covered by soft tissue in six cases, and firm occlusion was observed only in three animals. In contrast, in group B (with application CsA) the firm occlusion of the operative defect was proved in all cases, and no inflammatory response occurred around the site of the implanted material. It must be mentioned that no inflammatory response could be found in any of the animals (group A or group B).

### 3.2. Histological Evaluation

#### 3.2.1. Middle and Inner Ear Histology

**Figure 2:**
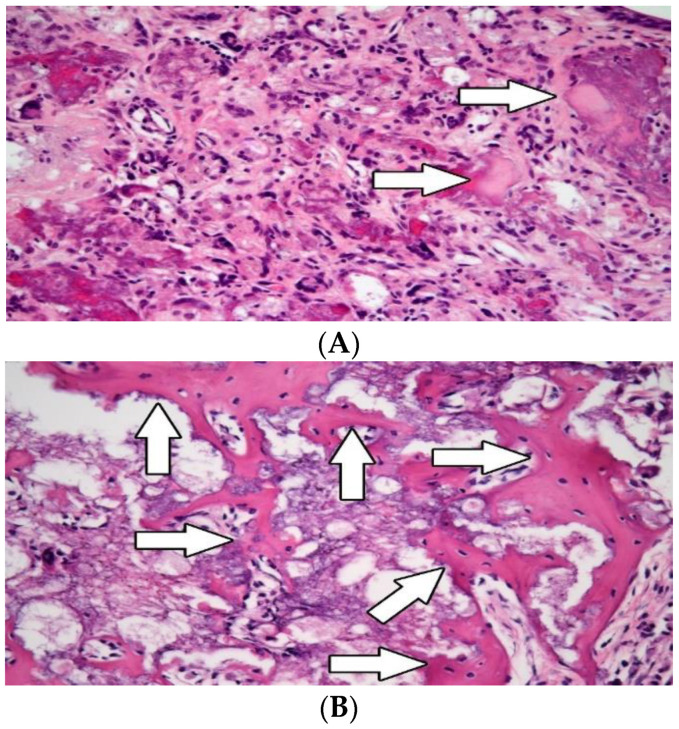
Histological examination of the tympanic bulla. The hydroxyapatite was seen in the tissue as a foreign material (white arrows) bubbly violet in direct contact with newly formed trabeculae of immature materiale woven bone. Compare the amount of newly formed woven bone between group A (without application of CsA; (**A**)) and group B (with application of CsA; (**B**))—white arrows. Hematoxylin-eosin, original magnification 400×.

Microscopic examination of the tympanic bulla showed a significantly higher incidence of newly formed bone trabeculae in group B in contrast to group A (Figure 2A,B). Furthermore, the histological evaluation showed a significantly improved repair of damaged bone tissue in group B.

The histological examination of the cochlea showed a vital neuroepithelium without any abnormalities in the inner ear in all samples.

#### 3.2.2. Osteogenesis

In group A, osteogenesis occurred at a lower rate with a mean ratio of new bone formation of 25,041 μm^2^, $\tilde{x}$ = 16,438 μm^2^, in 1 mm^2^ of implanted material (*p* < 0.0001). In group B, the new bone formation was significantly higher, the mean ratio was 808,172 μm^2^, $\tilde{x}$ = 874,776 μm^2^, in 1 mm^2^ of implanted material. The average volume of new immature bone in the temporal bone defect was 3% in group A compared with 81% in group B (*p* < 0.0001) (Table 1).

#### 3.2.3. Angiogenesis

**Figure 3:**
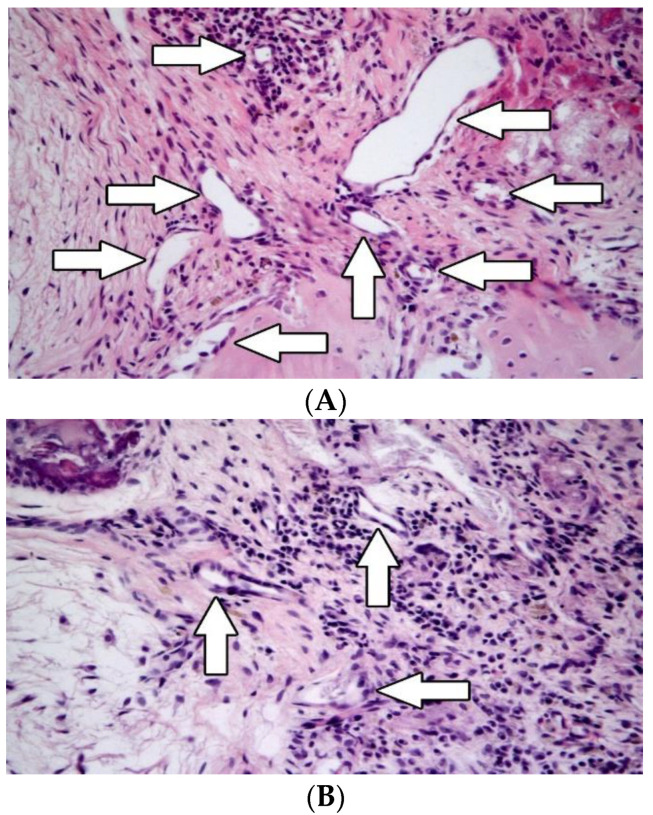
Angiogenesis was assessed by counting lumina of newly formed small blood vessels (white arrows). It was higher in group A (without application of CsA; (**A**)) compared with group B (with application of CsA; (**B**)). Hematoxylin-eosin, original magnification 400×.

Angiogenesis was observed in both groups. In group A, the mean number of blood vessels was 9, $\tilde{x}$ = 9 (*p* = 0.0152). In group B, the mean number of small blood vessel lumina was 7 in an area of 0.283 mm^2^, $\tilde{x}$ = 5. (Table 1 and Figure 3).

#### 3.2.4. Inflammation

**Figure 4:**
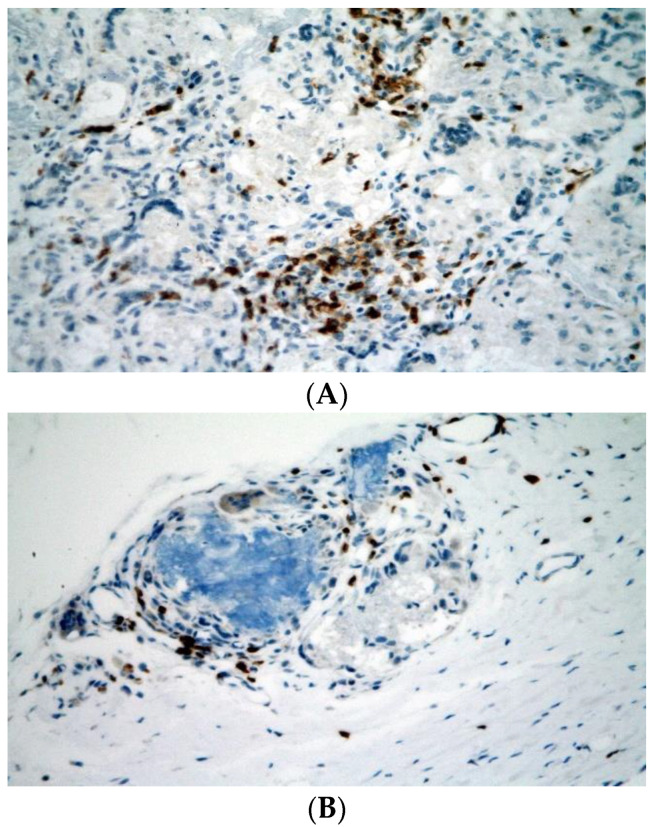
Their numbers were higher in group A (without application of CsA; (**A**)) compared with group B (with application of CsA; (**B**)). CD3-positive T-lymphocyte are recognized by brownish staining of cell membrane and cytoplasm. Their numbers were higher in group A (without application of CsA; (**A**)) compared with group B (with application of CsA; (**B**)) Immunohistochemistry, original magnification 400×.

**Figure 5:**
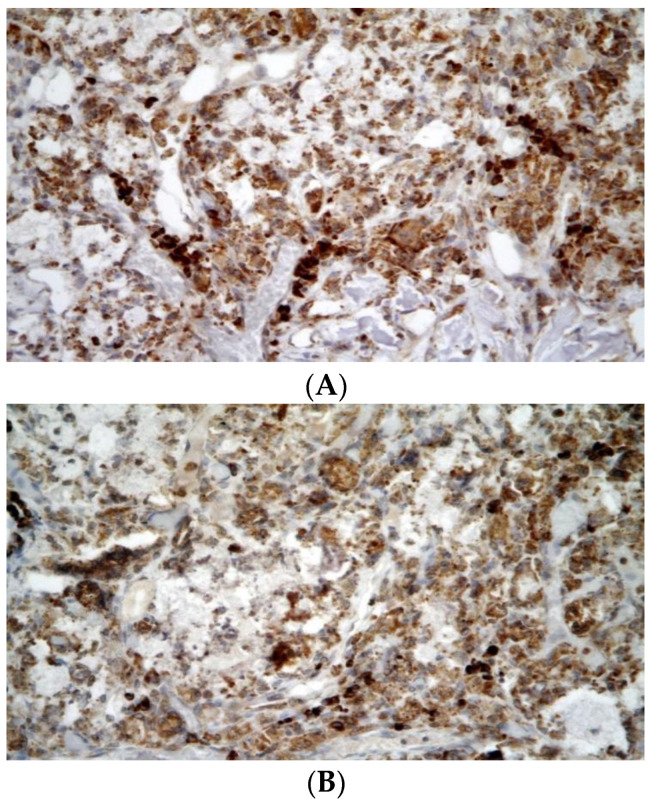
TRAP-positive cells were present in both group A (without application of CsA; (**A**)) and group B (with application of CsA; (**B**)) in roughly similar amounts. Their exact counting was not possible due to higher non-specific background. Immunohistochemistry, original magnification 400×.

Immunohistochemical examination proved the presence of CD3^+^ T-lymphocytes in the temporal bones of both groups. The mean number of CD3^+^ T-lymphocytes was 60 in group A and 16 in group B (*p* = 0.0057) (Table 1 and Figure 4A,B). TRAP^+^ cells were observed in all specimens (Table 1 and Figure 5A,B). CHAE+ neutrophils, CD20+ B-lymphocytes, and eosinophils were absent in all specimens.

### 3.3. Radiological Evaluation

In group A, the areal bone density was estimated in 394 HU ($\bar{x}$ = 390 HU) and “hot spot” bone density in 958 HU ($\bar{x}$ = 960 HU). In group B, the bone density in the area of the implanted material was estimated in 1473 HU ($\bar{x}$ = 1470 HU) and “hot spot” bone density in 1643 HU ($\bar{x}$ = 1640 HU). Compared with group A, both area and “hot spot” bone density were significantly higher in group B (*p* < 0.0001, *p* = 0.00276 resp.) (Table 2).

**Figure 6:**
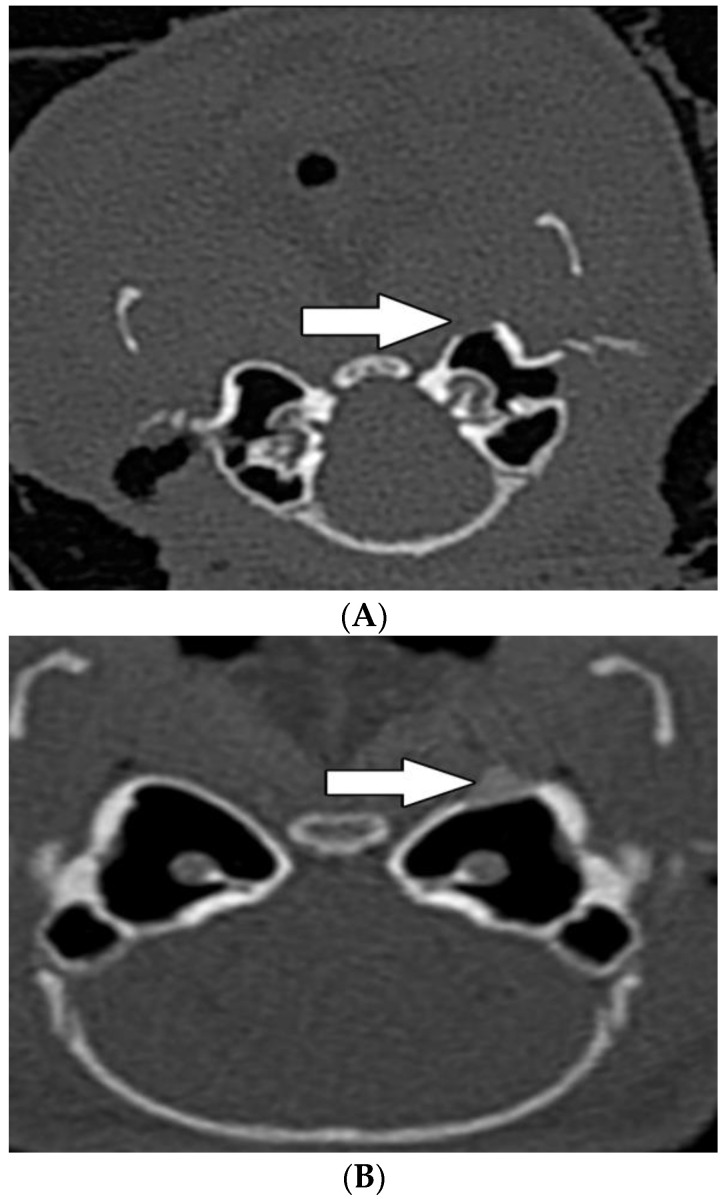
Radiological examination by HRCT at the level of the tympanic bulla, 60 days after implantation of MSCs in group A (**A**) and group B (**B**). Radiological examination by HRCT at the level of the tympanic bulla, 60 days after implantation of MSCs in group A (**A**) and group B (**B**). White arrows show the place of the implanted biomaterial into the bone defect.

Thickening of the middle ear mucosa was observed in three animals, in one of them the tympanic bulla was filled by fluid (Table 2). CT scans in both groups did not reveal pathological changes in the inner ear (Figure 6A,B).

## 4. Discussion

The treatment of middle ear cholesteatoma sometimes requires surgical treatment performing a canal wall down mastoidectomy (CWD). However, the procedure has a number of complications associated with anatomical changes in the external and middle ear, which are also involved with the loss of self-cleansing ability and chronic infections. There are no suitable surgical procedures allowing the complete closure of the mastoid cavity and the anatomical normalization of the middle and external ear [12,13].

Current research seeks to expand the treatment options for reconstructive surgery after CWD mastoidectomy. In this regard, biocompatible materials such as tricalcium phosphate, hydroxyapatite [14,15] or MSCs can be implemented [16]. 

The present study focused on the repair of the temporal bone after surgery using a hydroxyapatite scaffold loaded with an MSC suspension. Tissue glue was added to create the optimal consistency of the material. The obtained results from previous studies [17] demonstrate an improved regenerative capacity thanks to the combination of hydroxyapatite, MSCs, and immunosuppressive treatment of CsA. 

However, what is the role of CsA in the treatment of temporal bone reconstruction? The comparison of the non-CsA-treated group (A) and CsA-treated group (B) shows an interesting result. In the CsA-treated group (B), the new bone formation is significantly higher. The histological finding is consistent with higher bone density in CT scans and clinical explorations of tympanic bulla. Several studies show MSCs do not require an immunosuppressive treatment because of their own immunosuppressant capacity [9] involving the role of multiple cell receptors (e.g., ICAM-1, Gal- 9, PD-L1, TIGIT, CD200, and CXCR4) [10]. However, in vitro studies prove a beneficial interaction between MSCs and immunosuppressive drugs [18]. It has also been shown that CsA promotes the survival of MSCs in vivo [19]. There is evidence suggesting that CsA can affect the circulation and properties of bone-marrow-derived MSCs (BM-MSCs) [8] and promote the survival of MSCs in vivo; therefore, the combination of MSCs and CsA is a plausible alternative to improve treatment results [19,20]. All considered, it is still necessary to confirm the need of immunosuppressant drugs in combination with MSCs to increase the immunomodulating effect, which may have both positive and negative effects on the engraftment process [20,21].

Yet, the question remains regarding the effect of CsA on the surrounding cells and the added immunomodulatory effect to that of MSCs. In this study, the macroscopic exploration of the tympanic bulla showed a firm occlusion of the operative defect in the anterior wall in the CsA group in all cases. There was no inflammatory reaction around the site of the implanted material. This observation is supported by previous reports demonstrating the effect of CsA on immune cells [22]. However, the migration of T-lymphocytes and macrophages could be caused by the paracrine effect of MSCs [23], an important characteristic of these cells associated with the inhibition or activation of certain immune cells, thereby influencing the overall response to tissue damage and repair [24].

The positive effect of CsA on immunomodulation and tissue repair must be further verified. In vivo experiments have shown that CsA affects the immune response and the cells involved in repair, mostly by suppressing the adverse inflammatory reaction caused by neutrophil granulocytes [25,26].

Past studies have documented the contribution of MSCs to the formation of new blood vessels by releasing angiogenic factors [27]. The absence of new blood vessels often causes the failure of bone repair applications due to insufficient nutrient supply. Successful bone grafts depend upon early vascularization promoting the ingrowth of osteogenic reparative cells into the composite implant in critical bone defects [16]. From this point of view, thorough research regarding the function of CsA is essential, including its ability to inhibit angiogenesis, the disruption of vascular homeostatic mechanisms, and vasculopathy-associated chronic rejection [28]. MSCs, on the other hand, display a beneficial paracrine effect and can induce angiogenesis [23,29]. The latter effect was observed in the results of our experiments, in which a significant number of small blood vessels was detected during the histological analysis after one month of hBM-MSCs-based treatment [11].

MSCs’ plasticity and osteogenic differentiation potential constitute the major reasons for their inclusion in bone tissue repair procedures [5,30]. This osteogenic capacity has been described in several studies, although it was not made clear if their results are due to the presence of implanted MSCs [6,14]. This study resolved this question in a positive manner, in the sense that the implemented model allowed the MSCs to survive for at least two months after using a hydroxyapatite carrier. 

However, the question of whether CsA and its immunomodulatory effect enables a longer MSC survival and improved treatment remains unanswered. In essence, CsA directly affects the inflammatory response and inhibits GVHD [18,19,22]. However, it is not possible to clearly separate the immunomodulatory effect of CsA on MSCs and the body’s own response concerning GVHD. Therefore, further research is still needed in this regard. The afore statement notwithstanding, the present study confirmed the need of an immunosuppressant drug, i.e., CsA, and its positive effect on temporal bone reconstruction in guinea pigs.

## 5. Conclusions

The results presented hereby demonstrate that CsA promotes MSC survival in vivo. In our study, the reconstruction of a temporal bone defect with hBM-MSCs was found to require an immunosuppressant drug to improve the treatment´s outcome. This finding may be of fundamental importance for the use of hBM-MSCs in combination with hydroxapatite in temporal bone reconstruction after cholesteatoma surgery.

## Figures and Tables

**Table 1:** Histopathological evaluation of the temporal bone, comparing group A and group B.

	Group A (Mean Values ± SEM)	Group B (Mean Values ± SEM)	Statistical Significance
New bone formation (in 1 mm^2^)	25,041 (SEM ± 10,259)	808,172 (SEM ± 48,834)	*p* = 0.000041
New bone formation (volume %)	2.4 (SEM ± 1)	81 (SEM ± 5)	*p* = 0.000041
Angiogenesis	9 (SEM ± 2.4)	7 (SEM ± 1.6)	*p* = 0.0152
CD3	60 (SEM ± 5.9)	16 (SEM ± 6.3)	*p* = 0.00573
CHAE	0	0	
CD20	0	0	

**Table 2:** Radiological examination by HRCT of the temporal bone, comparing group A and group B.

	Group A (Mean Values ± SEM)	Group B (Mean Values ± SEM)	Statistical Significance
Areal density (HU)	394 (SEM ± 73)	1473 (SEM ± 146)	*p* = 0.00001
Hot spot density (HU)	958 (SEM ± 126)	1643 (SEM ± 119)	*p* = 0.00276
Mucosal thickening	3	1	*p* = 0.576

## Data Availability

Further information regarding this study will be made available by the corresponding author upon request.
